# Influence of thickness and surface conditioning on fracture resistance of occlusal veneer

**DOI:** 10.1186/s12903-023-02932-0

**Published:** 2023-05-03

**Authors:** Noha Essam, Hassan Soltan, Ahmed Attia

**Affiliations:** 1 Department of Fixed Prosthodontics, Faculty of Dentistry, Mansoura university, El Gomhouria St, Mansoura, Dakahlia Governorate Egypt; 2 Faculty of Engineering, Mansoura university, Dakahlia Governorate, Egypt

**Keywords:** Occlusal veneer, Fracture resistance, Surface treatment, Thickness, lithium disilicate, CAD/CAM

## Abstract

**Background:**

The purpose of the current study was to assess the impact of restoration thickness, surface conditioning and the interaction between them on the fracture resistance of CAD/CAM fabricated lithium disilicate occlusal veneers.

**Methods:**

A total of 42 maxillary molars were prepared to receive CAD/CAM fabricated lithium disilicate occlusal veneer either with 0.5 mm (n = 21) or 1 mm (n = 21) thickness. Each main group was divided into 3 subgroups (n = 7), according to surface treatment, HF acid (HF-1, HF-0.5), acidulated phosphate fluoride (APF-1, APF-0.5) and Monobond etch & prime (MON-1, MON-0.5). Multilinik N (Ivoclar-Vivadent) adhesive resin cement was used for bonding according to the manufacturer instructions. One hour after bonding, specimens were stored in water bath for 75 days followed by cyclic loading fatigue for 240,000 cycles to simulate clinical situation. Finally, specimens were fractured under compressive load in (N) using a universal testing machine. Two and one-way ANOVA and Post Hoc Tukey test were used for statistical analysis.

**Results:**

The means ± SD (N) fracture load for each group were calculated. MON-1 group showed the highest fracture load (1644.7 ± 155.3) followed by HF-1 group (1514.6 ± 212.5). Meanwhile, APF-0.5 showed the lowest fracture load (962 ± 249.6).

**Conclusion:**

CAD/CAM fabricated lithium disilicate occlusal veneers can be used with a thickness of 0.5 mm instead of conventional crowns. Monobond etch & prime is recommended as a surface treatment for CAD/CAM fabricated lithium disilicate occlusal veneer due to biological hazards of Hydrofluoric acid.

## Background

Tooth surface loss (TSL) is a complex system that has a multi-etiology which is a combination of mechanical (attrition, abrasion, abfraction) or chemical (erosion) [1]. Mostly TSL patients presented at the clinic complaining of functional impairment, loss of vertical dimensions, esthetic impairment or sensitivity [2]. Because of the biological risk of full-crown preparation, such as vitality loss and the need for endodontic treatment over time, full-crown preparation is not recommended [3]. These concepts nowadays is replaced by minimal intervention strategies which integrates prevention, remineralization and minimal invasion for the placement and replacement of restorations [4]. Due to this, occlusal veneers have been developed for the prosthetic restoration of eroded teeth using minimally invasive techniques and conservative preparation [1, 5–7].

Lithium disilicate glass ceramics have a wide range of clinical applications due to its optical properties, mechanical behavior, ease of fabrication, etchable and can be adhesively cemented which enabled the minimal invasive concept [8, 9]. One of the factors that positively affecting the fracture resistance of the ceramic restoration is its increased thickness, But the modern minimally invasive concepts of restorative and prosthetic dentistry often support the use of thin occlusal restorations [10–17]. Many previous studies concerned various thicknesses of lithium disilicate with a promising fracture resistance values [10, 13–16]. However 0.5 mm thickness was debatable among many studies [11, 12, 17]. Hence, the minimum thickness allowed for lithium disilicate occlusal veneers is still in short supply and need more investigation.

Effective bonding of the ceramic surface is a critical step for the clinical success of indirect ceramic-bonded restorations [18]. The lithium disilicate surface changed mechanically by acid etching in order to encourage the roughness of ceramic surface so that luting resin cement can get through and penetrate into the pores, in addition to chemical bonding by a silane coupling agent. Acids that used for silicate etching include hydrofluoric acid (HF), acidulated phosphate fluoride (APF), ammonium hydrogen difluoride [19].

Hydrofluoric acid (HF) etching creates a deep porous structure by removing and dissolving the glassy phase matrix. Pore size rely upon the etching time and etching concentration [19]. Previous studies revealed that HF acid etching might negatively affect the lithium disilicate strength and that the average surface roughness was negatively correlated to the mechanical strength [20]. However, the weakening effect of HF acid etching is time dependent [20]. HF acid is a potentially dangerous substance. It was found that it can cause serious tissue damage with low concentration. A 5% HF acid can cause dermal absorption and superficial skin damage when exposed for 3 min. With higher concentration it can penetrate into deeper tissue and cause severe damage so other etching materials are used [21].

Due to its low hydrofluoric acid content and few fluoride ions, acidulated phosphate fluoride (APF) works more superficially on the ceramic surface [22]. While HF acid is not appropriate for intra oral use, APF is used in orthodontics bracket bonding on porcelain crown and intraoral ceramic repair [23].

Silanization enhanced the ceramic surface bond strength between the ceramic and the resin. It was reported that the application of silane and a layer of resin luting agent improve the strength of glass ceramic due to crack bridging [24]. Silane molecules incorporating the cracks, and polymerization shrinkage of the resin cement enhance the apparent strength of cemented ceramics by putting the molecules together, rather than away from each other, as a consequence, cracks would not be able to open freely [25].

The former bonding method for silicate ceramics requires more steps and takes longer time. HF has been substituted in a new product with ammonium polyfluoride, which also contains silane, combining the two surface treatments and simplifying the bonding process [26]. Ammonium polyfluoride has been proven to be more biocompatible, secure, and less aggressive than HF acid [27].

The overall load-bearing capacities of all-ceramic crowns could decrease after combined cyclic stress, moist and thermal cycling [28]. It has been stated that water storage softens the polymer of resin matrix and induce hydrolysis of the interfacial silane coupling agent [29, 30]. Furthermore, mechanical failure of dental restorations occurs after several years of use, reflecting fatigue rather than acute overload [31].

Comparative studies on the effect of thickness and surface treatment and their interaction on the fracture resistance of machinable lithium disilicate are scarce. Therefore, the present study was carried out to evaluate the influence of different thickness and surface treatment and their interaction on fracture resistance of occlusal veneer. The null hypothesis of the present study was that neither surface treatment nor veneer thickness could influence fracture load of CAD/CAM fabricated lithium disilicate occlusal veneers.

## Methods

### Materials

Materials used in this study are showed in Table (1).

**Table 1:** Showing the materials used in this study

Material	Specification	Manufacturer	LOT number	Chemical composition
IPS e.max CAD	Lithium disilicate glass ceramics	IvoclarVivadent AG, Schaan, Liechtenstein	S25999	SiO_2_ (57 – 80%), Li_2_O (11. % – 19%), K_2_O (0.0 – 13%), P_2_O_5_ (0.0 – 11.0%), ZrO_2_ (0.0 – 8.0%) ZnO (0.0 – 8.0%), Al_2_O_3_ (0.0%– 5. %), MgO (0.0–5.0) and coloring oxides (0.0–8.0)
IPS Ceramic Etching Gel	Hydrofluoric acid 5%	IvoclarVivadent AG, Schaan, Liechtenstein	Y50956	< 5% hydrofluoric acid (HF)
Monobond N	Universal primer	IvoclarVivadent AG, Schaan, Liechtenstein	Y29210	Alcohol solution of silane methacrylate, phosphoric acid methacrylate, and sulphide methacrylate
Monobond Etch & Prime	Self etching glass ceramic primer	IvoclarVivadent AG, Schaan, Liechtenstein	Y27773	Alcoholic-aqueous solution of ammonium polyfluoride, silane methacrylate, and colorant.
Mirage porcelain etchant kit	Acidulated phosphate fluoride	Mirage, Kansas, USA		4% acidulated phosphate fluoride
Multilink N	Dual cure resin cement	IvoclarVivadent AG, Schaan, Liechtenstein	Y26001	The monomer matrix is composed of dimethacrylate and HEMA. The inorganic filler includes barium glass, ytterbium trifluoride, and spheroid mixed oxide. Particle size is 0.25-3.0 μm. Total inorganic filler volume is 40%
kemapoxy 150 3D	Epoxy resin	CMB, Giza, Egypt		Two components, solvent free, non-pigmented liquid epoxy resin
Meta Etchant	Phosphoric acid	Meta Biomed, Germany		Phosphoric acid, H_2_O, xanthan gum
Silaxil	Condensation silicon rubber base	Lascode, Italy		Base paste: siloxane prepolymer with terminal hydroxyl group and filler Catalyst paste: tin actoate and ortho alkyl silicate
Multilink N Primer A and B	Dental adhesive	IvoclarVivadent AG, Schaan, Liechtenstein		Primer A is an aqueous solution of initiators. Primer B contains HEMA, phosphonic acid and methacrylate monomers.

### Specimens preparation

To detect the difference of 5% with an effect size of 1.72, a sample size of 7 specimens was needed in each group. The sample size was calculated by using G* (version 3.0.10); Germany.

Forty-two sound maxillary molars, extracted from healthy individuals due to periodontal reasons, were collected. The teeth were thoroughly examined under a magnifying loupes with 5X magnification (Univet, Italy). With the use of a caliber, molars that significantly surpassed the average dimensions of 12 ± 2 mm buccolingual and 10 ± 2 mm mesiodistal width were eliminated. The teeth were disinfected for 72 h by being placed in a 1% chloramine-T solution after soft tissue remains were removed. In an incubator (BTC, Model: BT1020, Cairo, Egypt), the teeth were kept in distilled water at a temperature of 37 °C ± 1 °C and the water was renewed regularly every 5 days during the study period. Roots of each molar were embedded and fixed vertically in transparent epoxy resin blocks (kemapoxy 150 3D, CMP international, Egypt).

### Molars preparation

**Fig. 1:**
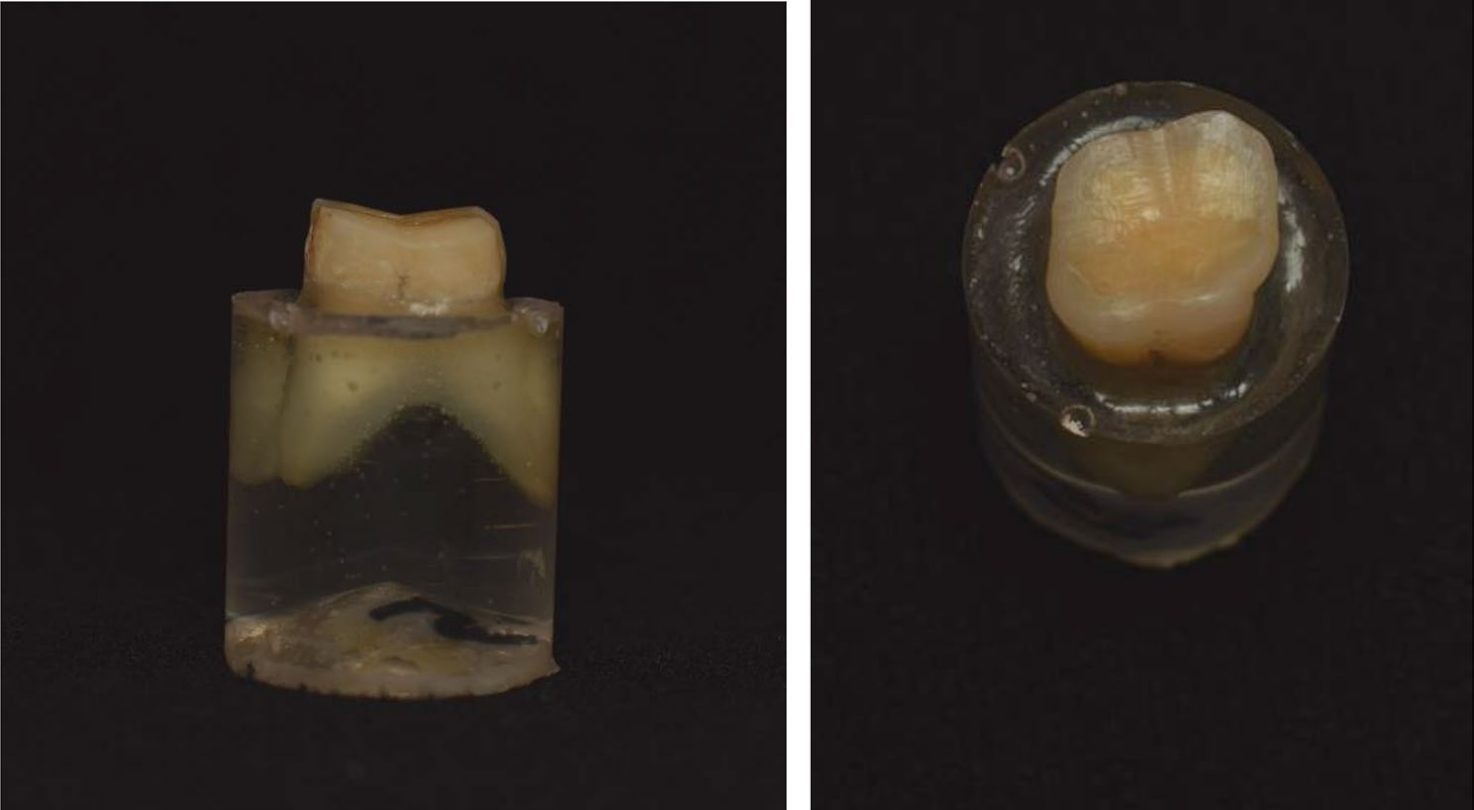
Photograph showing preparation design

Molars were divided into two main test groups (n = 21), according to thickness of occlusal veneer, either 0.5 or 1 mm thickness. Therefore, occlusal surface of molars was prepared either with 0.5 or 1 mm occlusal reduction to mimic tooth wear (Fig. 1). The reduction amount was evaluated by a previously fabricated silicon index at least three times during preparation. Preparation was conducted using freehand technique by the researcher using high speed handpiece under constant copious water coolant irrigation.

### Fabrication of occlusal veneers

**Fig. 2:**
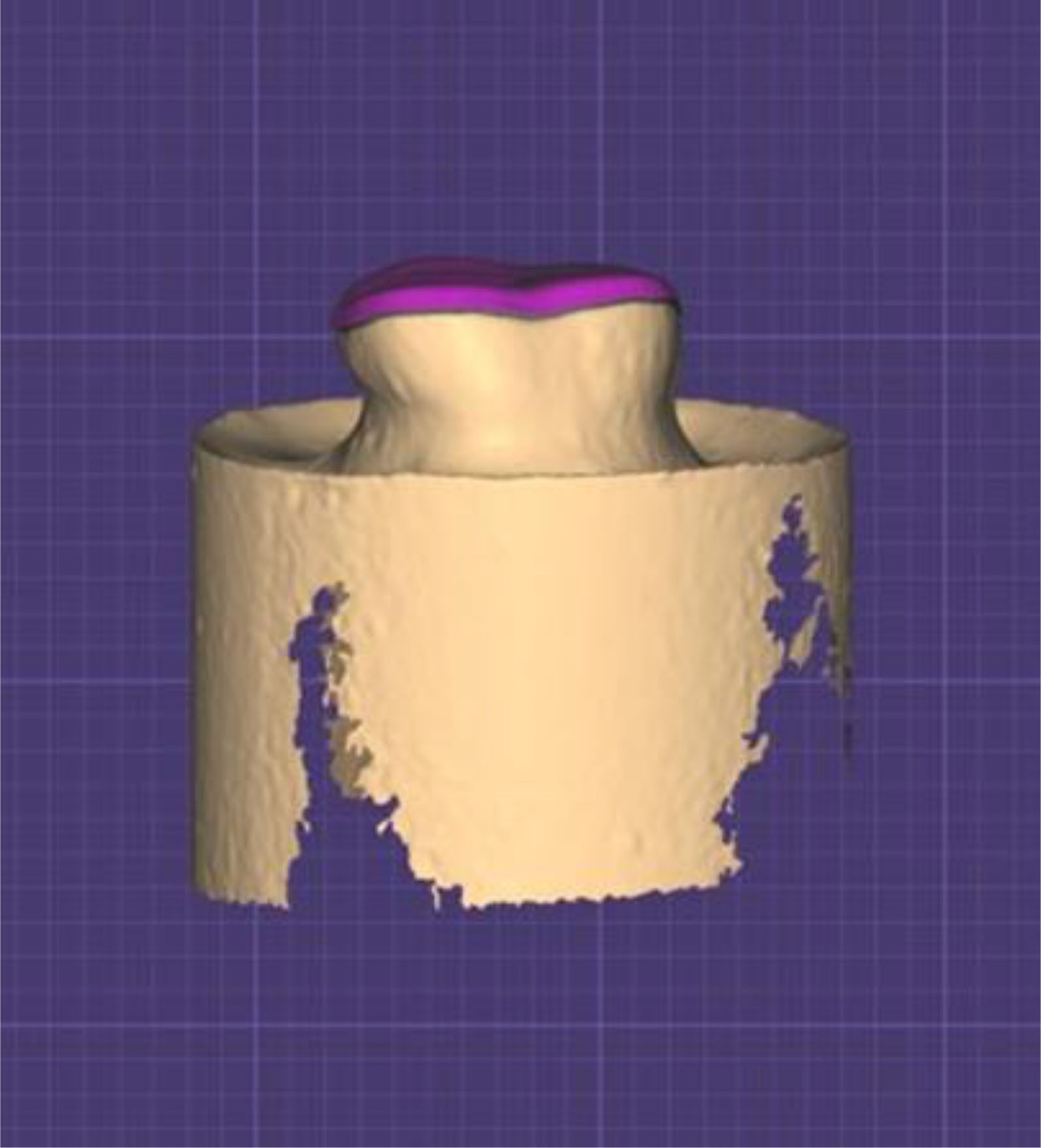
Photograph showing CAD design of the veneer

To achieve the opaque surface needed for CAD scanner (Ceramill Map 400, Amann Girrbach, Koblach, Austria), silver powder (CERCON, DeguDent GmbH, Germany) was applied to the prepared tooth surface. By using design software (EXOCAD DentalCAD GmbH, 64,293-Darmstadt, Germany), occlusal veneers were designed in a way uniforming the thickness of the restoration [15]. Thickness was set according to main test groups (n = 21 ) either 0.5 or 1 mm. Thickness was adjusted to be the same from cusp and fissure (Fig. 2) Occlusal veneers were wet-milled from lithium disilicate blocks (IPS e.max CAD, Ivoclar Vivadent AG, Schaan, Liechtenstein) by using a 5-axis milling machine (CERAMILL MOTION 2, Amann Girrbach AG, Herrschaftswiesen, Austria).

### Surface treatment of lithium disilicate occlusal veneers

Each main test group (n = 21) was subdivided to three subgroups (n = 7) according to surface treatment of occlusal veneer as follow:

- Subgroup (HF): Occlusal veneers were etched with hydrofluoric acid (HF) followed by universal primer (Monobond N) application.
- Subgroup (APF): Occlusal veneers were etched with acidulated phosphate fluoride (APF) followed by universal primer (Monobond N) application.
- Subgroup (MON): Occlusal veneers were conditioned with self-etching primer (Monobond etch & prime).

#### HF acid etching and Monobond N according to manufacturer instructions

5% HF acid (IPS Ceramic Etching Gel, Ivoclar Vivadent) was applied for 20 s. to the bonding surface of the veneer. The surface was irrigated with water for 60 s. Veneers were dried with strong stream of water and oil free air for 10 s. A thin coat of universal primer (Monobond N, Ivoclar Vivadent) was applied with a microbrush and allowed to react for 60 s. Remaining excess was dispersed with strong stream of air.

#### APF acid etching and Monobond N according to manufacturer instructions

APF acid etch (MIRAGE, USA) was applied for 2 min to the bonding surface of the veneer. The surface was rinsed off with water stream for 60 s. Veneers were dried with strong stream of water and oil free air for 10 s. A thin coat of universal primer (Monobond N) was applied with a microbrush and allowed to react for 60 s. Any remaining excess was dispersed with strong stream of air.

#### Self-etching primer (Monobond etch & prime) according to manufacture instructions

Monobond etch & prime (Ivoclar Vivadent) was applied using a microbrush to the bonding surface of the veneer. It was agitated into the surface for 20 s and allowed to react for another 40 s. The surface was rinsed off with water stream until green color was removed. Veneers were dried with strong stream of water and oil free air for 10 s.

### Tooth surface conditioning

Selective etching technique was used. Phosphoric acid 37% (Meta etchant) was applied to the enamel surface for 15–30 s, then rinsed with vigorous stream of water for at least 5 s. Finally, etched molars were dried with water and oil free air stream till chalky white appearance was reached. The two primer liquids Multilink N Primer A and B (Ivoclar Vivadent) were mixed and applied according to manufacturer instructions. There was no necessity for light-curing because the Primer self-cure.

### Bonding of the veneer

**Fig. 3:**
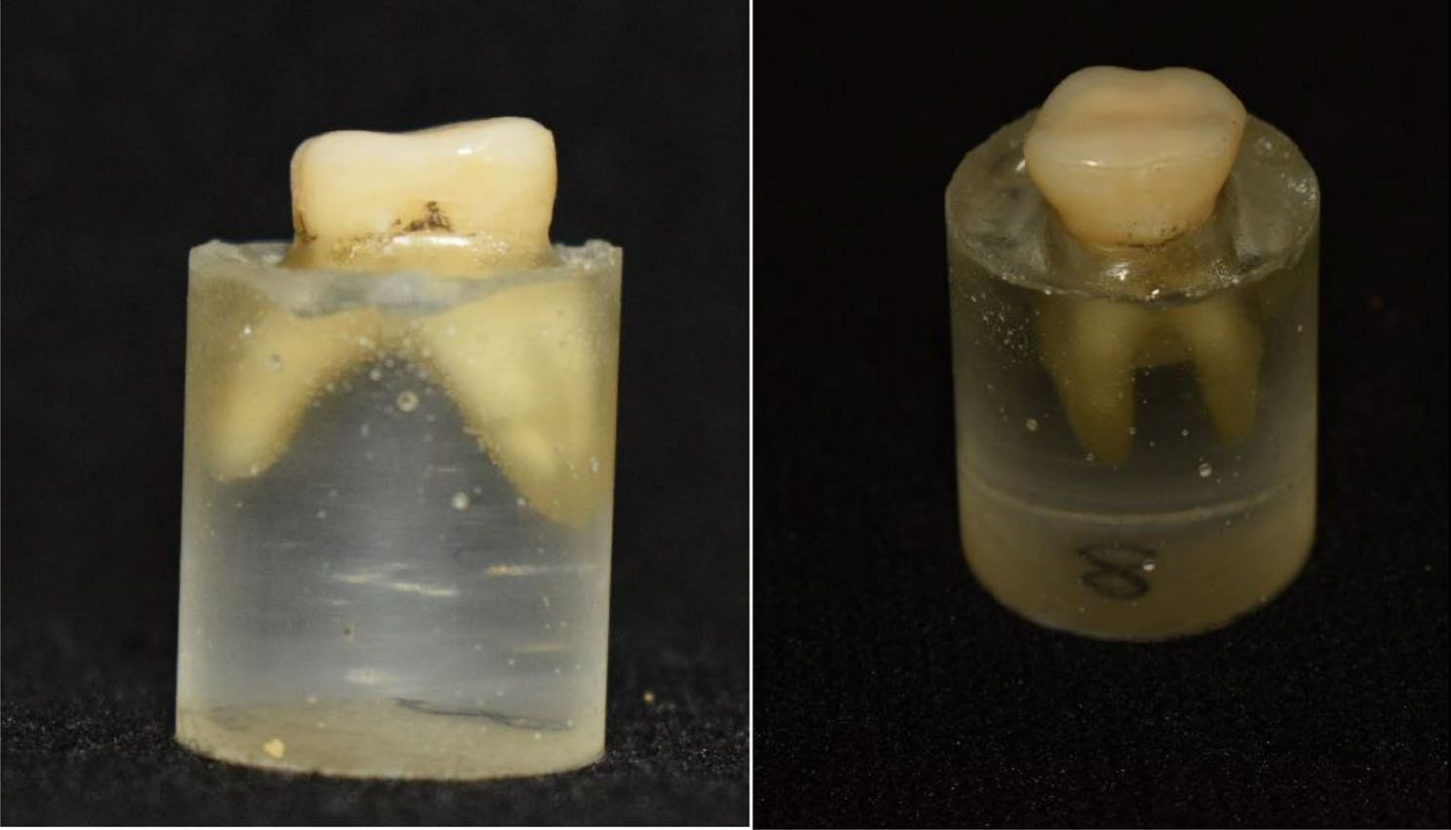
Occlusal veneers bonded to maxillary molars

Bonding was done using adhesive resin cement (Multilink N, Ivoclar Vivadent) according to the manufacturer instructions. The specimen was secured to a specially designed device with lever system to obtain load of 5 kg on the occlusal veneer/tooth assembly during bonding. The bonded assembly was kept for 5 min under the static load [32, 33] (Fig. 3).

### Artificial aging

#### Water storage

One hour after cementation, all specimens were stored in water bath at 37^o^C for 75 days in waterproof plastic pots.

#### Cyclic loading fatigue

**Fig. 4:**
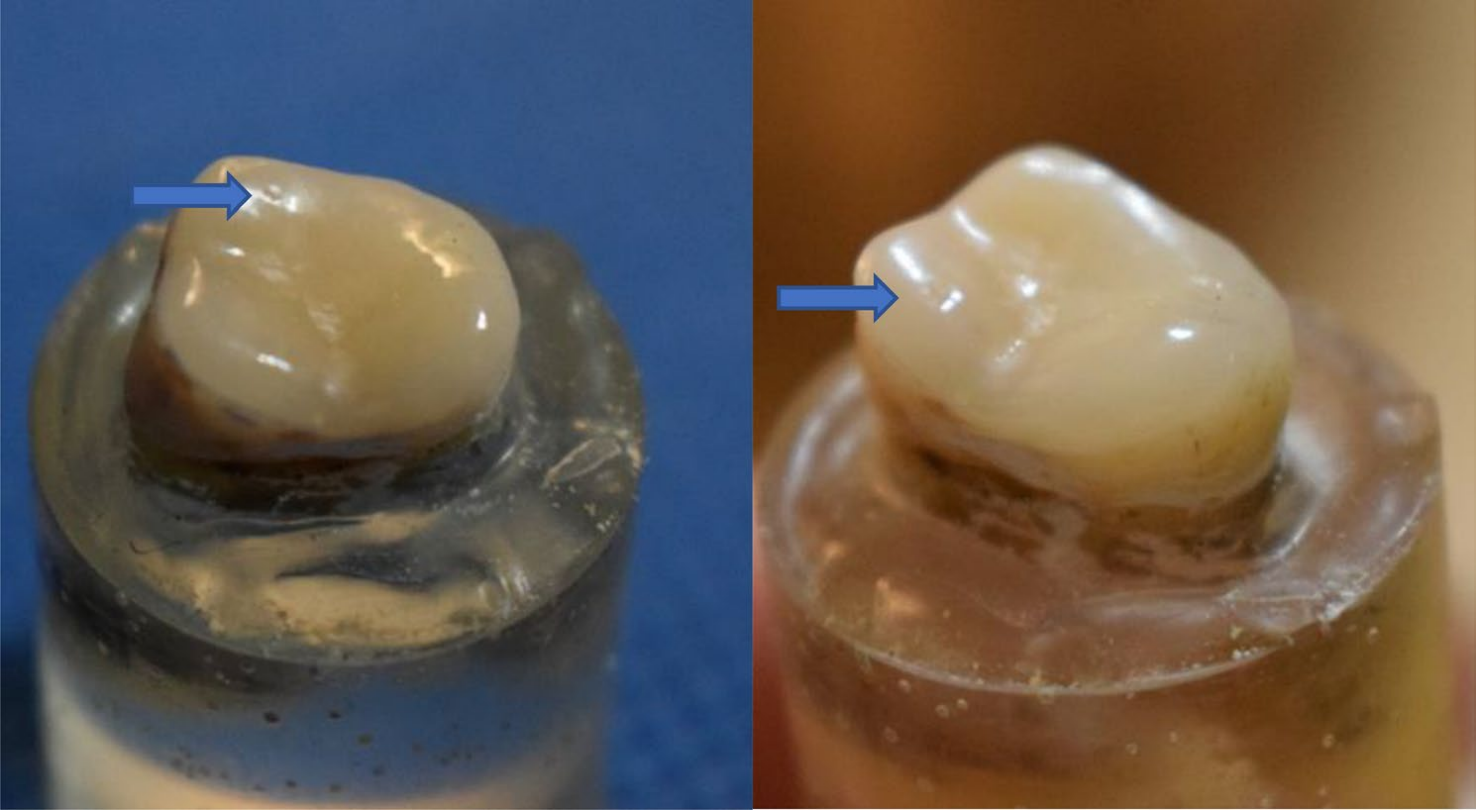
Photograph showing cyclic loading indentation on occlusal veneer

Cyclic loading fatigue was conducted using a deliberately designed custom-made cyclic loading machine (Department of biomaterials, Faculty of Dentistry, Alex university). Samples were exposed to 240,000 repeated mechanical cycles with opposing mandibular molars to simulate condition for one-year in the oral environment [34]. Using a weight of 49 N and a loading frequency of 1.7 Hz, circumstances similar to those during normal chewing and swallowing were replicated [35]. After cyclic loading, the specimens were visually inspected to see whether any cracks were present, which would indicate failure. After cyclic loading, indentations appeared on occlusal veneer surface (Fig. 4)

### Fracture load test

Specimens were loaded by compressive force till first failure either crack or fracture using a Universal testing machine (Instron 3345, USA) (Bluehill Universal software, Instron, USA). A 5 mm metal sphere was positioned in the central fossa and in contact with the cuspal inclines. Direct contact was made between the metal sphere and the cuspal inclines. Fracture load was applied at 0.5 mm/min crosshead speed. The first crack formation was recorded for each specimen in Newton (N).

### Failure analysis

The failed specimens were examined using Binocular optical microscope to determine the mode of failure. Assessment criteria were categorized according to Al-Akhali et al. (2017) [14] into:

I = Extensive crack formation within the restoration.

II = Adhesive fracture.

III = Cohesive fracture.

IV = Longitudinal fracture of the restoration and the tooth.

Further evaluation of representative specimen of each failure pattern was done under high magnification using Scanning Electron Microscope (SEM).

### Statistical analysis

Statistical analyses were done by Social Package for Statistical Science (SPSS) software version 25.0. Statistical analyses were done with two-way ANOVA and serial one-way ANOVAs at each level of the study followed by Post Hoc Tukey (HSD) test. Statistical significance (P) is significant if (P < 0.05).

## Results

### Fracture resistance test

**Fig. 5:**
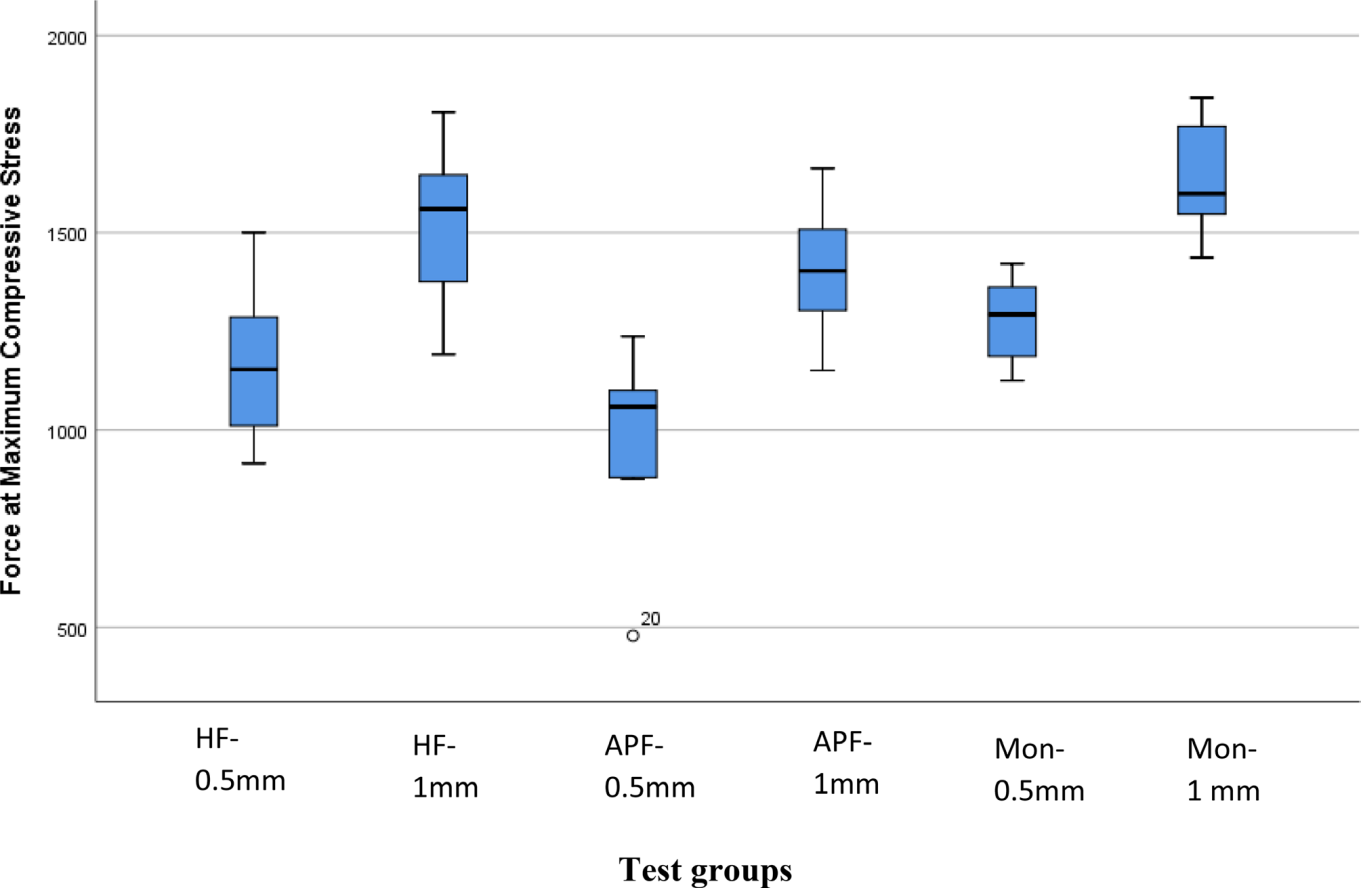
Box Plots showing means fracture load of tested groups in (N)

The collected data were tabulated, coded then analyzed using the computer program statistical package for social science (SPSS) version 25. Mean fracture load of all test groups were demonstrated with box plots in (Fig. 5).

Mean fracture load (N) of all test groups were compared with a 2-factor ANOVA model (Table 2), including the following factors: surface treatment, veneer thickness, and their interactions. The overall ANOVA F-test was significant (P < 0.0001), indicating difference in mean fracture load across at least one of the factors. Veneer thickness (P < 0.0001), and surface treatment of the occlusal veneer (P = 0.002) were significant. However, their interaction was not significant (P = 0.785).

**Table 2:** Showing two-way ANOVA test at different levels of the study

	Type III Sum of Squares	df	Mean Square	F	Sig.
Veneer Thickness	1569959.468	1	1569959.468	43.036	< 0.0001
Surface Treatment	539808.627	2	269904.313	7.399	0.002
Veneer Thickness * Surface Treatment	17767.838	2	8883.919	0.244	0.785
Error	1313296.470	36	36480.458		
Total	77555775.380	42			
Corrected Total	3440832.403	41			

Further analyses with serial one-way (ANOVA)s were used to test the effect of each factor independently. P value was (0.036) when surface treatment was considered (Table 3), and P value was (0.0001) when veneer thickness was analyzed (Table 4).

**Table 3:** Showing one way ANOVA test considering different surface treatment of veneers

Maximum compressive strength	Sum of Squares	df	Mean Square	F	Sig.
Between Groups	539808.627	2	269904.313	3.628	0.036
Within Groups	2901023.776	39	74385.225		
Total	3440832.403	41			

**Table 4:** Showing one way ANOVA test considering different veneer thickness

Maximum compressive strength	Sum of Squares	df	Mean Square	F	Sig.
Between Groups	2127535.933	5	425507.187	11.664	0.000
Within Groups	1313296.470	36	36480.458		
Total	3440832.403	41			

Post Hoc Tukey (HSD) test was used for pairwise comparison between different tested groups (Table 5). Considering veneer thickness with the same surface treatment, there were statistically significant differences between the following test groups: (HF-1 mm, HF-0.5 mm, P = 0.018), (MON-1 mm, MON-0.5 mm, P = 0.011) and (APF-1 mm, APF-0.5 mm, P = 0.001). Considering surface treatment at the same thickness, there was statistically significant differences between test groups (MON-0.5 mm, APF-0.5 mm, P = 0.042). However, there were no statistically significant differences between the other test groups at the same thickness (P > o.o5).

**Table 5:** Showing means ± SD fracture load of tested groups in (N): Post Hoc Tukey HSD test (p ≤ 0.05)

groups	Mean ± SD	HF-0.5 mm	HF- 1 mm	APF-0.5 mm	APF-1 mm	MON- 0.5 mm	MON- 1 mm
HF-0.5 mm	1166.2 ± 211		0.02*	0.4	0.2	0.9	0.001*
HF-1 mm	1514.6 ± 212.5	0.02*		0.000*	0.9	0.2	0.8
APF- 0.5 mm	962 ± 249.6	0.4	0.000*		0.001*	0.04*	0.000*
APF-1 mm	1405.8 ± 172.7	0.2	0.9	0.001*		0.8	0.2
MON- 0.5 mm	1277 ± 114	0.9	0.2	0.04*	0.8		0.01*
MON- 1 mm	1644.7 ± 155.3	0.001*	0.8	0.000*	0.2	0.01*	

HF = hydrofluoric acid

APF = acidulated phosphate fluoride

MON = Monobond etch and prime

(*) indicating statistically significant difference

### Failure pattern analysis

**Fig. 6:**
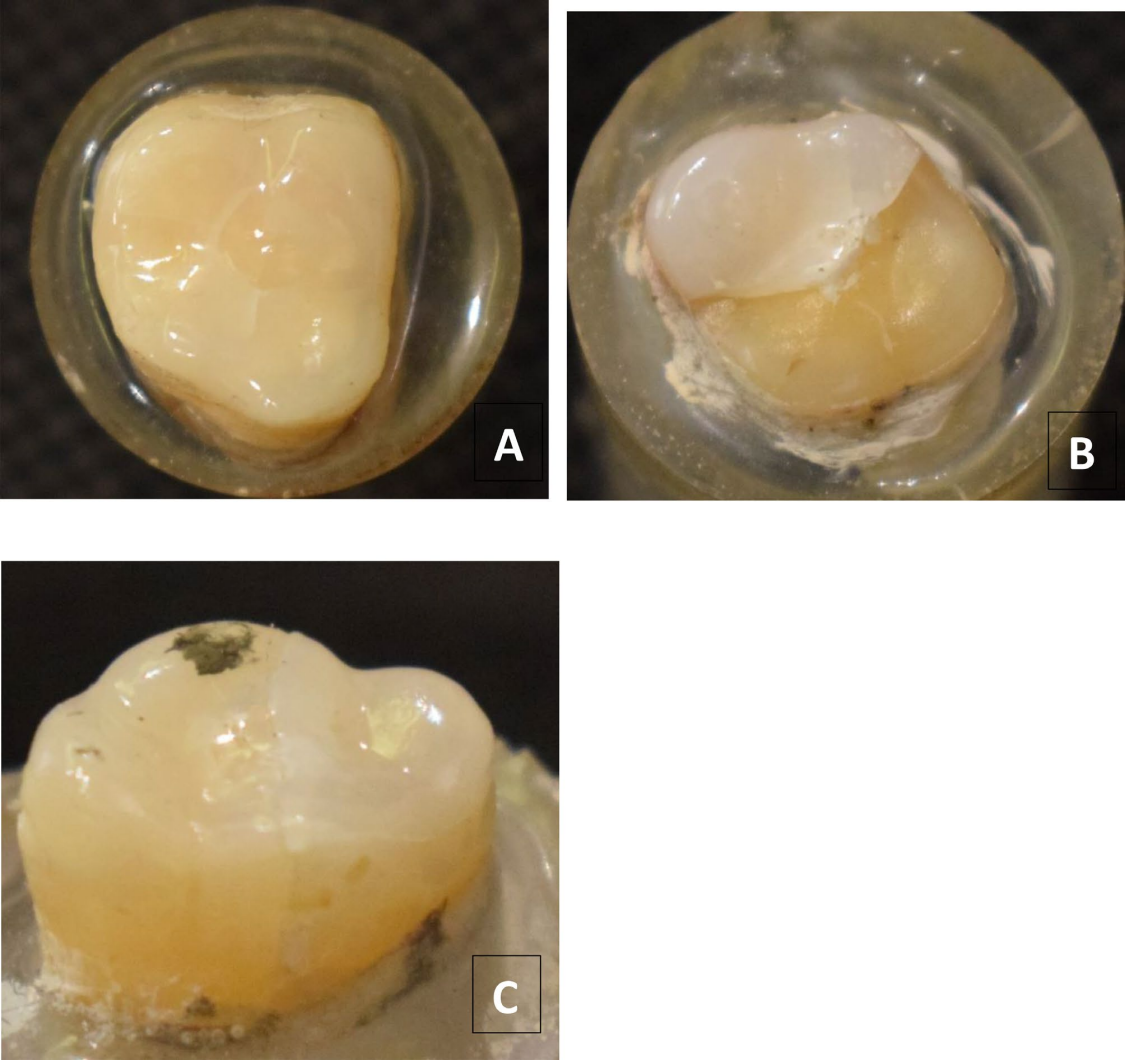
Failure patterns. **A**; failure mode (I) (extensive crack formation within the veneer without separation from the tooth), **B**; failure mode (II) (cohesive fracture within the restoration with separation from the tooth), **C**; failure mode (III) (longitudinal fracture of the restoration and the tooth)

Al-Akhali et al. (2017) [14] criteria was modified according to the findings of this study. No adhesive failure was found. The modified criteria: Mode I = Extensive crack formation within the veneer without separation from the tooth (Fig. 6A). Mode II = Cohesive fracture within the veneer with separation from the tooth (Fig. 6B). Mode III = Longitudinal fracture of the restoration and the tooth (Fig. 6C).

Failure mode was mainly extensive crack formation within the veneer without separation from the tooth (failure mode I) (27 specimens), followed by longitudinal fracture of the restoration and the tooth (failure mode III) (10 specimens), then Cohesive fracture within the veneer with separation from the tooth (failure mode II) (5 specimens). HF and MON groups showed failure mode I and III. APF groups showed failure mode II and III.

### Scanning electron microscope (SEM) examination

**Fig. 7:**
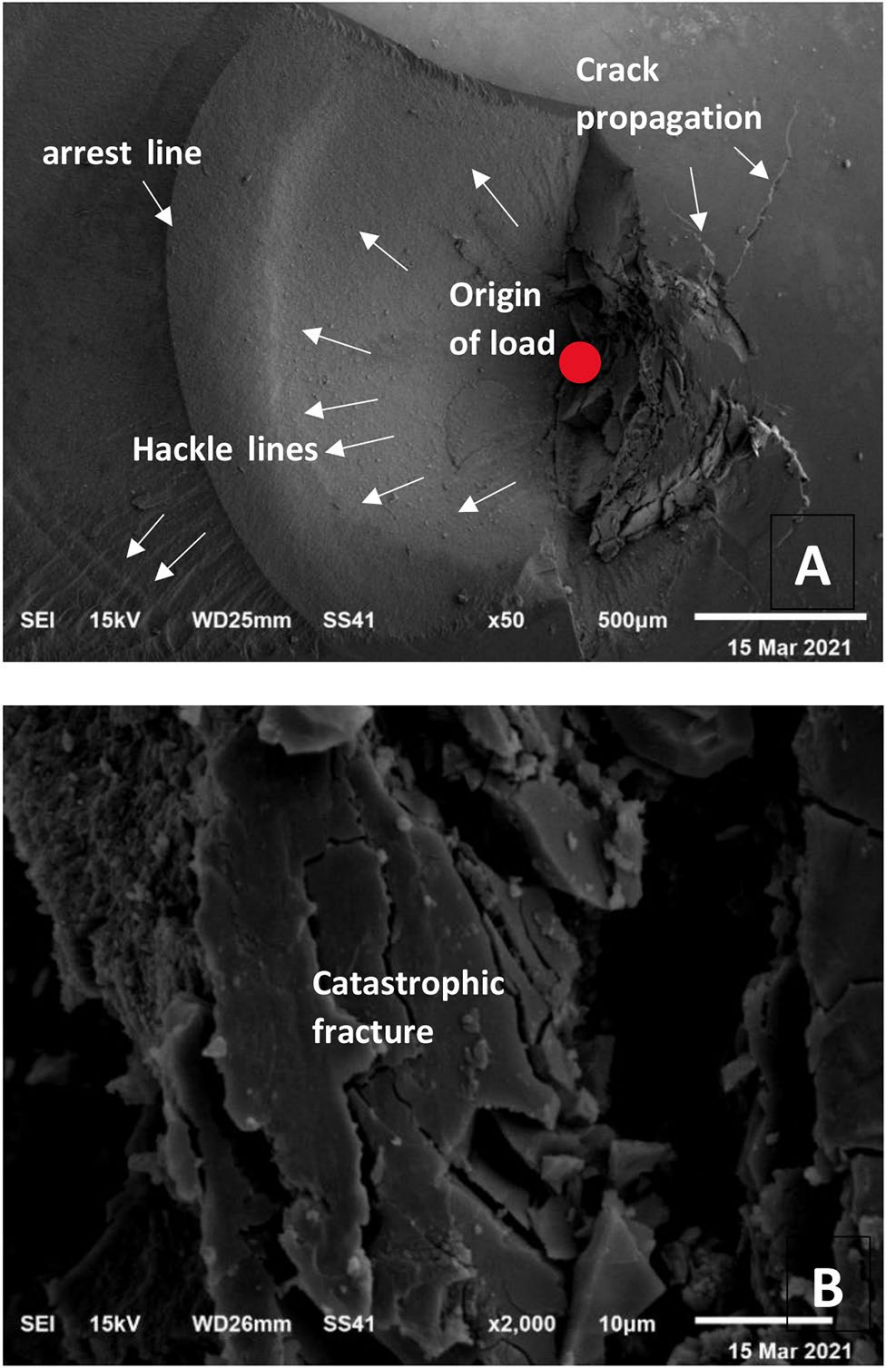
SEM micrograph showing failure mode (I) (extensive crack formation within the veneer without separation from the tooth): **(A)** x50 magnification, **(B)** x2000 magnification

**Fig. 8:**
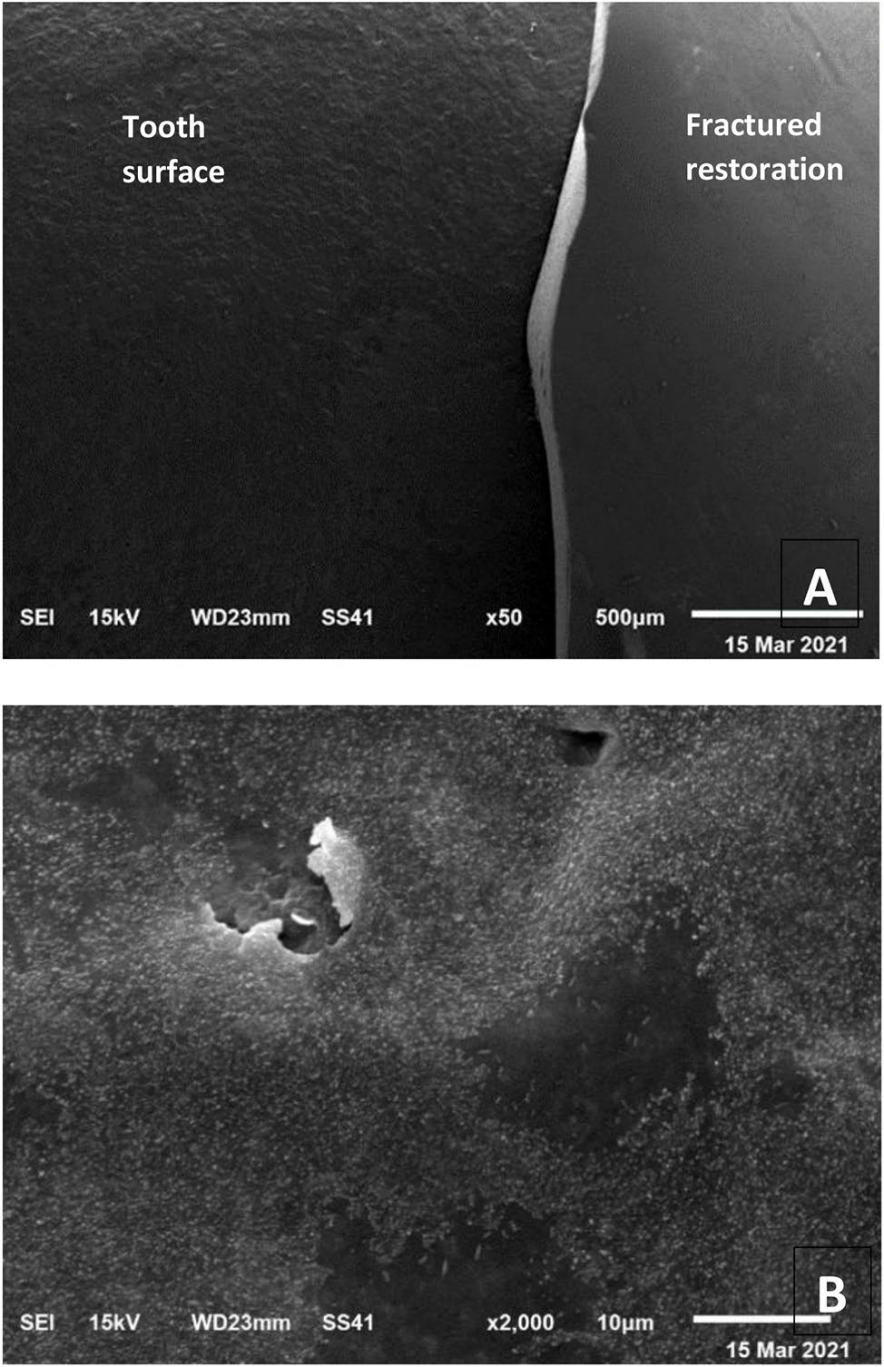
SEM micrograph showing failure mode (II) (cohesive fracture within the restoration with separation from the tooth): **(A)** x50 magnification, **(B)** x2000 magnification

**Fig. 9:**
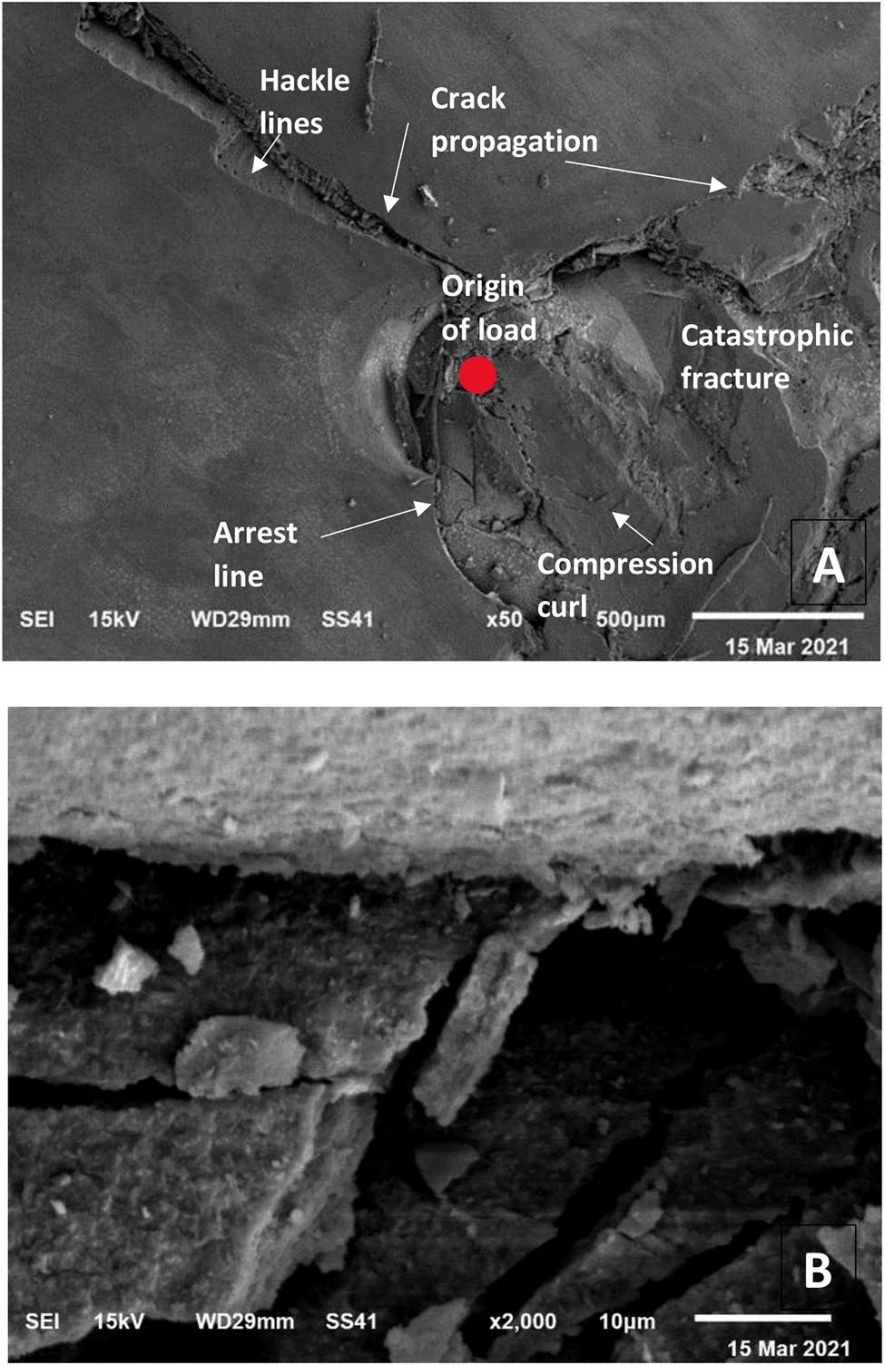
SEM micrograph showing failure mode (III) (longitudinal fracture of the restoration and the tooth): **(A)** x50 magnification, **(B)** x2000 magnification

Further evaluation of failure modes was done by SEM (Figs. 7, 8 and 9). fractographic analysis was done. Failure started at the load origin. Hackle lines, arrest lines and direction of crack propagation were observed.

## Discussion

The hypothesis of the present study was that neither surface treatment nor veneer thickness could influence fracture load of CAD/CAM fabricated lithium disilicate occlusal veneers. However, the results of the present study rejected both hypotheses. Fracture load of 1 mm thickness occlusal veneer was significantly higher than 0.5 mm thickness occlusal veneers regardless of surface treatment. Also, surface treatment affected fracture load of occlusal veneers at the same thickness.

In order to simulate intraoral conditions, specimens were stored in water for 75 days followed by cyclic loading fatigue for 240,000 cycles with opposing natural molars to simulate one year clinically. The aging procedures of the specimens did not lead to any failure of the restorations. Ioannidis et al. (2019) [15] and Maeder et al. (2019) [36] applied load of 49 N at 1.7 Hz loading frequency for 1’200’000 cycles of dynamic loading, which was reported in literatures to simulate 5 years of clinical service. All specimens survived the cyclic loading and thermal cycling. In accordance with this, 0.5 mm thickness occlusal veneers fabricated from CAD/CAM lithium disilicate seems to be promising for mouth rehabilitation in case of TSL in the molar area. However, normal masticatory forces can reach higher values than 49 N. In the posterior region, they can range from 400 to 800 N [37]. Taking this into consideration, clinicians should be aware of the values which are achieved by fracture resistance test.

The results of this invitro study revealed that maximum fracture load mean value for HF-0.5 group differed from HF-1 group. Test group with 1 mm thickness showed higher fracture load. However, fracture load of HF-0.5 group yielded maximum fracture load at higher value than the masticatory force, which ranges from 400 to 800 N in the molar area [37]. Ioannidis et al. (2019) [15] and Maeder et al. (2019) [36] reported similar results. On the other hand, Schlichting et al. (2011) [11] did not recommend the use of lithium disilicate in 0.6 mm thickness in patient with excessive occlusal force. Also, Sasse at al. (2015) [12] reported 610 N fracture load of 0.3–0.6 mm group and recommended the use of lithium disilicate occlusal veneers with a thickness of 0.7 to 1 mm. Differences in results between the present study and other studies might be due to different research parameters that cannot be unified for all researches.

In this study, when the occlusal veneers were treated with Monobond etch & prime 1 mm thickness showed higher results than 0.5 mm group. Similarly, Baldissara et al. (2019) [38] studied Monobond etch & prime treated lithium disilicate occlusal veneers with different thickness (0.5, 0.8, and 1.2 mm). Survival was significantly influenced by the restoration thickness. Thicker restorations exhibited a higher survival rate. However, in the present study MON-0.5 group fracture load yielded maximum fracture load at higher value than the masticatory force in the molar area.

Similarly, there was significant difference between APF-1 and APF-0.5 groups. Unlike HF-0.5 group and MON-0.5 group, APF-0.5 mean fracture load was 962.05 ± 249.56 N. This value is close to the masticatory force in the molar area when bruxism existed (800 N). So APF treated 0.5 mm occlusal veneers fabricated from lithium disilicate is not recommended for patients with parafunctional oral habits such as bruxism and clenching.

In the current study, it was noticed that MON groups showed the highest mean fracture load for 0.5 and 1 mm thickness, followed by HF groups. The least values were reported for APF groups for both thicknesses. In the English language literature to the best of our knowledge, rare previous studies investigated the fracture load of lithium disilicate under compressive load with different surface treatments especially APF. Many previous studies declared the weakening effect of HF on lithium disilicate [21, 39]. HF etching increased ceramic roughness, even for periods as short as 20 s which is the time recommended by the manufacturer. The authors explained the decrease in flexure strength by the amount of the glass phase involving the lithium disilicate crystals. This might explain the higher mean fracture load of MON test groups than HF test groups in this study.

In terms of fracture load, the present study found that there was no significant difference between Monobond etch & prime and HF & Monobond N surface treatment at the same thickness. Also, in terms of bond strength, Maier et al. (2019) [40], Román-Rodríguez et al. (2017) [26] and Wille et al. (2017) [21] found that tensile bond strength for Monobond etch & prime was equivalent to HF and silane. However, HF acid is a potentially dangerous substance [21]. It was found that it can cause serious tissue damage with low concentration used in dental field [21]. Furthermore, it has been proven that ammonium polyfluoride is more biocompatible and secure than HF [26]. Thus, to avoid health hazards of HF in the dental office, Monobond etch & prime is recommended to be used.

It has been proven that adequate bonding between the ceramic-cement-tooth complex surely increased the ceramic strength [3]. In the present study APF treated samples showed the less fracture load mean values in 0.5 mm samples and 1 mm samples. This might be explained by the fact that APF produced shallow irregularities with deposits precipitation in a way compromising the bond strength between ceramic surface and resin, while ceramic surface gets its strength from strong bond of ceramic-cement-tooth complex. Once this bond was compromised, ceramic strength decreased. These results might be supported by the results of Canay et al. (2001) [22] and Della Bona et al. (2002) [18]. However, other research [23, 41] found that regardless the less roughness produced by APF, bond strength was not compromised. This disagreement with results of this study might be due to different etchant concentration, different etching time, and different etched materials.

Mode of failure of APF samples was mainly fracture with separation of the veneer from the underlying tooth structure unlike other samples that was mainly extensive crack formation in the restoration only or in the restoration tooth complex without debonding of occlusal veneers. SEM topography revealed catastrophic fracture of occlusal veneers in HF and MON groups, while in APF groups part of the restoration de-bonded after fracture showing etched tooth surface with remaining cement particles. Crack formation without debonding form of failure indicated that the bond of restoration-cement-tooth complex was stronger than the force applied. Meanwhile, separation of APF treated samples indicated that the bond at veneer/cement/prepared tooth interfaces was affected [42].

Regarding surface treatment, there was significant difference between APF-0.5 group and MON-0.5 group. However, there was no significant difference between other surface treatment groups at the same thickness. Hence, at 1 mm thickness any surface treatment can be used. But when it comes to 0.5 mm thickness, APF is not recommended and Monobond etch & prime is preferred.

Regarding the interaction between thickness and surface treatment, there was not statistically difference in the current study (p = 0.785). By far in the English language literature, we could not find previous studies investigating the interactive relation between thickness and surface treatment, therefore further research should be conducted.

Limitation of this in-vitro study could be the fact that bonded specimens were stored in water only. However, in the clinical situation the bonded restoration is exposed to saliva and other beverages. In addition, bonded restoration intraorally subjected to variation in temperature, therefore, thermal cycling is needed in further investigation. Also, it is an invitro study so long-term clinical study should be conducted to support the results of this invitro study.

## Conclusion

Within the limitation of this invitro study, the following conclusions were drawn:

1. Thickness of CAD/CAM fabricated lithium disilicate occlusal veneers affected the fracture load.
2. 0.5 mm thickness occlusal veneer could be used in molar area with proper surface treatment.
3. Monobond etch & prime is recommended for surface treatment to avoid the health hazards of HF in the dental office.
4. The interaction between thickness and surface treatment of occlusal veneers did not affect the fracture load.

## Data Availability

The data sets used and/or analyzed during the current study are available from the corresponding author upon reasonable request.

## Abbreviations

HF: Hydrofluoric acid
APF: Acidulated phosphate fluoride
TSL: Tooth surface loss
CAD/CAM: Computer aided design; computer aided manufacture
SEM: Scanning electron microscope
SPSS: Social Package for Statistical Science
